# TSLP production by dendritic cells is modulated by IL‐1β and components of the endoplasmic reticulum stress response

**DOI:** 10.1002/eji.201545537

**Published:** 2015-12-02

**Authors:** Matthew J. Elder, Steven J. Webster, David L. Williams, J. S. Hill Gaston, Jane C. Goodall

**Affiliations:** ^1^ Department of Medicine, University of Cambridge School of Clinical MedicineAddenbrookes HospitalCambridgeUK; ^2^ Department of Surgery, Center for Inflammation, Infectious Disease and Immunity, James H. Quillen College of MedicineEast Tennessee State UniversityJohnson CityTNUSA

**Keywords:** Dectin‐1, ER stress, IL‐1β, TSLP

## Abstract

Thymic stromal lymphopoietin (TSLP) produced by epithelial cells acts on dendritic cells (DCs) to drive differentiation of T_H_2‐cells, and is therefore important in allergic disease pathogenesis. However, DCs themselves make significant amounts of TSLP in response to microbial products, but little is known about the key downstream signals that induce and modulate this TSLP secretion from human DCs. We show that human monocyte derived DC (mDC) secretion of TSLP in response to *Candida albicans* and β‐glucans requires dectin‐1, Syk, NF‐κB, and p38 MAPK signaling. In addition, TSLP production by mDCs is greatly enhanced by IL‐1β, but not TNF‐α, in contrast to epithelial cells. Furthermore, TSLP secretion is significantly increased by signals emanating from the endoplasmic reticulum (ER) stress response, specifically the unfolded protein response sensors, inositol‐requiring transmembrane kinase/endonuclease 1 and protein kinase R‐like ER kinase, which are activated by dectin‐1 stimulation. Thus, TSLP production by mDCs requires the integration of signals from dectin‐1, the IL‐1 receptor, and ER stress signaling pathways. Autocrine TSLP production is likely to play a role in mDC‐controlled immune responses at sites removed from epithelial cell production of the cytokine, such as lymphoid tissue.

## Introduction

Thymic stromal lymphopoietin (TSLP) is a four‐helix bundle cytokine closely related to IL‐7, and was initially described as a lymphocyte growth factor 1. Subsequently, it has been shown to promote the development of T_H_2 immunity 2, 3, to be important in maintaining tolerance within the gut 4, 5, and to be a pathogenic factor in the development of atopic inflammation 6, 7 and inflammatory arthritis 8, 9. TSLP signals via a specific receptor composed of a unique TSLP receptor chain and the IL‐7 receptor α chain (IL‐7Rα) 10, leading to JAK‐STAT signaling 11, 12, 13, and downstream transcription of target genes.

TSLP potently activates dendritic cells (DCs), priming them to drive T_H_2‐cell differentiation 2, 10. However, in addition to being a target of TSLP, we and others have recently shown that human monocyte derived DC (mDCs) themselves produce TSLP in response to pattern‐recognition receptor (PRR) engagement 14, 15. Furthermore, DC‐derived TSLP has been shown to be functionally important in protection against intestinal inflammation by modifying T‐cell phenotype to promote the development of Foxp3^+^ T cells 16. Mechanisms that control autocrine production of TSLP by mDCs have not previously been characterized; thus the aim of this work was to determine the signals that induce and modulate human TSLP production by mDCs.

Among PRRs, dectin‐1 stimulation can induce TSLP mRNA expression in mDCs 15. Dectin‐1 recognizes β‐1,3 glucans (β‐glucans) on fungi and certain bacteria, initiating downstream signaling events that drive effector responses including phagocytosis 17, inflammasome activation 18, 19, 20, 21, and inflammatory cytokine release 22, 23, 24, important for antifungal immunity. Myeloid cells such as DC mediate these immune responses and are essential for resistance to systemic fungal infections in mice 25. Therefore continued understanding of signals that control DC functional responses to fungal infection is extremely important.

We have shown that synergistic signaling from PRR and endoplasmic reticulum (ER) stress greatly enhanced TSLP gene transcription in mDCs, similar to the effect of ER stress on IL‐23p19 expression 14. Although ER stress has previously been described as regulating cytokine secretion 14, 26, 27, 28, and hence influencing inflammatory and autoimmune diseases 26, 29, the effects on IL‐23p19 and TSLP were particularly striking 14. Therefore, we wished to determine the key signals that induce TSLP in human mDCs, and the pathways of the ER stress response that modulate TSLP production by mDCs.

The ER senses dangers to internal homeostasis and initiates the unfolded protein response (UPR) when the protein folding capacity of the cell is exceeded causing disruptions to normal protein folding mechanisms. The UPR is controlled by the three principal ER stress sensing proteins, inositol‐requiring transmembrane kinase/endonuclease 1 (IRE1α), protein kinase R‐like ER kinase (PERK), and activating transcription factor 6 14, 26, 27, 28, 30. These proteins control discrete yet interconnected signaling pathways that modulate specific genes involved in orchestrating cellular responses to ER stress. The UPR allows the cell to resolve ER stress via various mechanisms including PERK‐mediated translation shutdown via the phosphorylation of eukaryotic initiation factor 2 α (eIF2α); however, if these mechanisms fail, programmed cell death is initiated 31.

Here, we show that dectin‐1 signaling stimulates TSLP production from mDCs and that this is enhanced by signals from the ER stress sensing proteins, IRE1α, and PERK. In addition dectin‐1‐induced IL‐1β is required for optimal TSLP secretion; however, we show that dectin‐1‐induced TSLP requires independent, mutually exclusive signals emanating from the ER stress response and the IL‐1 receptor.

## Results

### β‐glucans induce TSLP in human mDCs, and require dectin‐1 signaling via Syk, NF‐κB, and p38 MAPK

**Figure 1:**
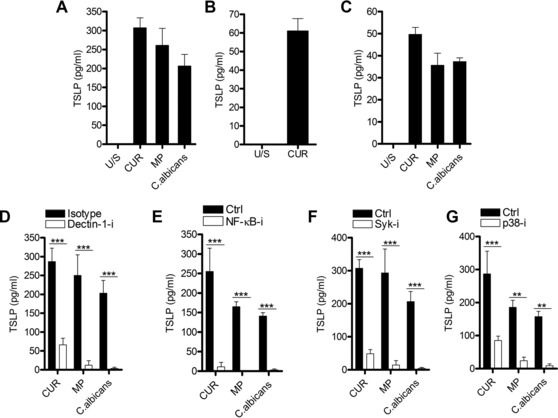
β‐glucans induce TSLP in human mDCs, and require dectin‐1 signaling via Syk, NF‐κB, and p38 MAPK. (A) Human mDCs were differentiated from CD14^+^ monocytes for 6 days using GM‐CSF and IL‐4 and were stimulated with the dectin‐1 agonists curdlan (CUR); β‐1,3 glucan MPs or heat‐killed *C. albicans* (MOI 2:1) for 24 h (*n* = 6 independent donors, presented as pooled data). (B) Human ex vivo CD1c^+^ DCs were isolated from PBMCs and were stimulated with CUR for 24 h (*n* = 2 independent donors, presented as pooled data). (C) Murine BMDCs were differentiated from cells isolated from mouse femurs for 7 days using 5% X63 conditioned media and IL‐4 and were stimulated with CUR, MP, and *C. albicans* for 24 h (*n* = 3 independent animals from a representative experiment, presented as pooled data). (D–G) Human mDCs were preincubated for 1 h with or without (D) anti‐dectin‐1 or isotype control (*n* = 5 independent donors, presented as pooled data), (E) Syk inhibitor (*n* = 6 independent donors, presented as pooled data), (F) NF‐κB inhibitor (*n* = 6 independent donors, presented as pooled data) or (G) p38 MAPK inhibitor (*n* = 5 independent donors, presented as pooled data) and were then stimulated with CUR, MP, or *C. albicans* for 24 h. (A–G) TSLP was measured in 24‐h cell culture supernatants by ELISA. Cumulative data are displayed as mean ± SEM. Statistical significance was calculated using (A) one‐way or (C–F) two‐way ANOVA with Bonferroni post‐tests (***p = 0.001, **p = 0.01).

Since previous work showed that the dectin‐1/TLR2 agonist zymosan was efficient at inducing TSLP expression in mDCs 15, we used three different β‐glucan preparations as dectin‐1 agonists: curdlan (a large particulate derived from *Alcaligenes faecalis*), smaller particulate β‐1,3 glucan microparticles (MPs) derived from *Saccharomyces cerevisiae*, and the hyphal yeast, *Candida albicans*. Dectin‐1 signaling stimulated via *C. albicans* or β‐glucan particle engagement induced TSLP secretion in immature mDCs (CD83^lo^, CD86^lo^, HLA‐DR^+^, Fig. 1A). The ability to secrete TSLP in response to dectin‐1 stimulation was not an artefact of the in vitro differentiation from monocytes, since ex vivo blood‐derived CD1c^+^ DC also demonstrated this property (Fig. 1B). Furthermore, murine BM‐derived DC (BMDCs) can secrete TSLP after β‐glucan stimulation (Fig. 1C).

Blocking dectin‐1 with a specific antibody (Fig. 1D), or employing a specific Syk inhibitor (Fig. 1E), potently inhibited TSLP production in response to all three stimuli. β‐glucan stimulation induced Syk phosphorylation (Tyr525/526) in mDCs with Syk inhibition preventing this phosphorylation event (Supporting Information Fig. 1A) and dectin‐1 neutralization had no effect on peptidoglycan‐induced IL‐1β (Supporting Information Fig. 1B).

Activation of NF‐κB 22, 32 and p38 MAPK 32 mediate dectin‐1‐induced inflammatory cytokine expression, and both NF‐κB 33, 34 and p38 MAPK 35 are required for TSLP induction in epithelial and stromal cells, respectively. In agreement with this, we found that β‐glucan‐stimulated mDCs induced both NF‐κB activation, measured by IκBα degradation (Supporting Information Fig. 1C) and p38 MAPK phosphorylation (Thr180 and Tyr182, Supporting Information Fig. 1D). Furthermore, *C. albicans* or β‐glucan‐induced TSLP required activation of both NF‐κB (Fig. 1F) and p38 MAPK (Fig. 1G) in mDCs; however, p38 MAPK inhibition did not affect IL‐23 secretion (Supporting Information Fig. 1E). This indicated that in human mDCs NF‐κB interaction with the TSLP promoter and signaling by p38 MAPK are requirements for dectin‐1‐induced TSLP.

### IL‐1β but not TNF‐α is required for dectin‐1‐induced TSLP secretion in mDCs

**Figure 2:**
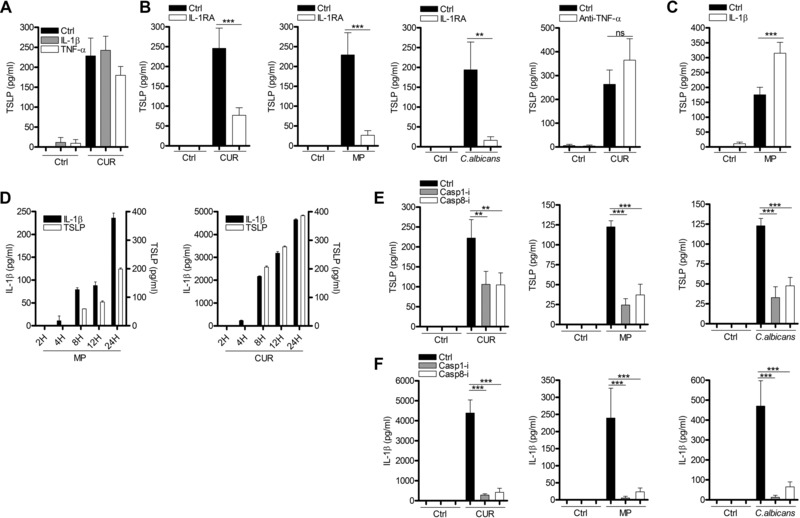
IL‐1β but not TNF‐α is required for dectin‐1‐induced TSLP secretion in mDCs. (A) Human mDCs were stimulated with CUR with or without IL‐1β or TNF‐α for 24 h (*n* = 3 independent donors, presented as pooled data). (B) mDCs were preincubated for 1 h with or without IL‐1RA or anti‐TNF‐α and were then stimulated with CUR (*n* = 8 independent donors, presented as pooled data), MP (*n* = 11 independent donors, presented as pooled data) or *C. albicans* for 24 h (*n* = 4 independent donors, presented as pooled data). (C) Human mDCs were stimulated with MP with or without IL‐1β for 24 h (*n* = 9 independent donors, presented as pooled data). (D) Human mDCs were stimulated with CUR or MP for 2, 4, 8, 12, or 24 h. (E, F) Human mDCs were preincubated for 1 h with or without caspase‐1 or caspase‐8 inhibitors and were then stimulated with CUR, MP or *C. albicans* for 24 h (*n* = 5 independent donors, presented as pooled data). (A–F) TSLP and IL‐1β were measured in 24‐h cell culture supernatants by ELISA. Cumulative data are displayed as mean ± SEM. Statistical significance was calculated using one‐way ANOVA with Bonferroni post‐tests (***p = 0.001, **p = 0.01).

Dectin‐1 signaling in DC induces IL‐1β 18, 19 and TNF‐α 32, 36 secretion in addition to TSLP. Both of these cytokines alone can induce epithelial cells 33, keratinocytes 37, fibroblasts 8, smooth muscle cells 38, and stromal cells 35 to secrete TSLP. In contrast to these reports, treatment of mDCs with IL‐1β or TNF‐α alone, induced negligible quantities of TSLP (Fig. 2A). Although the addition of IL‐1β alone failed to induce substantial TSLP secretion in mDCs, inhibition of IL‐1 signaling using the IL‐1 receptor antagonist (IL‐1RA) significantly reduced the quantity of TSLP induced by *C. albicans* or β‐glucans (Fig. 2B). Therefore, IL‐1β signaling is necessary but not sufficient for the induction of TSLP secretion by dectin‐1 agonists. In contrast, TNF‐α did not exhibit this property, as shown by the inability of a neutralizing anti‐TNF‐α antibody (Supporting Information Fig. 2) to modulate TSLP secretion induced by dectin‐1 agonists (Fig. 2B).

In contrast to curdlan, MPs are poor inducers of inflammatory cytokines such as IL‐6 and IL‐23 in DC 23, 24. We also show that compared to large β‐glucan particles, MPs are poor inducers of

IL‐1β secretion. Importantly, these small quantities of IL‐1β were essential for TSLP production, indicated by the profound loss of TSLP expression when IL‐1RA was present in mDCs culture with MP (Fig. 2B). To determine if the small quantities of IL‐1β induced by MP were suboptimal for TSLP secretion, we added additional recombinant IL‐1β. This augmented TSLP secretion, but only to an equivalent quantity detected using curdlan stimulation (Fig. 2C). The addition of IL‐1β to curdlan‐stimulated mDCs had no effect on TSLP secretion (Fig. 2A), presumably because there were saturating quantities of IL‐1β present. The potential for IL‐1β to augment TSLP expression is evident by the presence of detectable IL‐1β 4 h prior to TSLP secretion (Fig. 2D). Although IL‐1RA did not inhibit curdlan‐induced TSLP as potently as TSLP induced by the other dectin‐1 agonists (Fig. 2B), this may simply reflect that IL‐1RA is an inefficient antagonist and was unable to completely inhibit IL‐1β signaling in the presence of high concentrations of IL‐1β. These data suggest that even the small quantities of IL‐1β provided by MP exceed a critical threshold that enables substantial secretion of TSLP by mDCs.

Processing and secretion of IL‐1β requires inflammasome activation mediated by caspase recruitment and activation 18, 19, 20, 21. Previous work has described a role for both caspase‐1 18, 19, 20, 21 and caspase‐8 19, 21 in fungal‐induced IL‐1β; thus we used specific inhibitors of caspase‐1 and caspase‐8 to test for a requirement for caspase activation in *C. albicans* and β‐glucan‐induced TSLP. Activation of both caspase‐1 and caspase‐8 was required for dectin‐1‐induced TSLP (Fig. 2E), and IL‐1β (Fig. 2F). Furthermore, caspase‐1 KO BMDCs stimulated with *C. albicans* or β‐glucan showed reduced TSLP and IL‐1β (Supporting Information Fig. 3A and B). Our findings showed that dectin‐1‐induced inflammasome/caspase‐dependent IL‐1β is required for optimal TSLP production from mDCs; however, inflammasome‐independent signaling events are also required.

Although Syk, NF‐κB, and p38 MAPK signaling is required for IL‐1β expression, the addition of exogenous IL‐1β could not rescue TSLP expression in the presence of these inhibitors (Supporting Information Fig. 4A–F). Therefore, the reduction of TSLP expression in the presence of these inhibitors cannot be solely attributed to the loss of IL‐1β.

### Dectin‐1‐induced IRE1α and PERK regulate TSLP production by mDCs

We have previously presented microarray data showing that ER stress can augment PRR‐induced TSLP and IL‐23p19 in mDCs 14, though this did not apply to the vast majority of cytokines examined. The importance of crosstalk between signals from PRR and those emanating from the ER stress response in the production of cytokines by DC has been emphasized recently 14, 26, 27.

**Figure 3:**
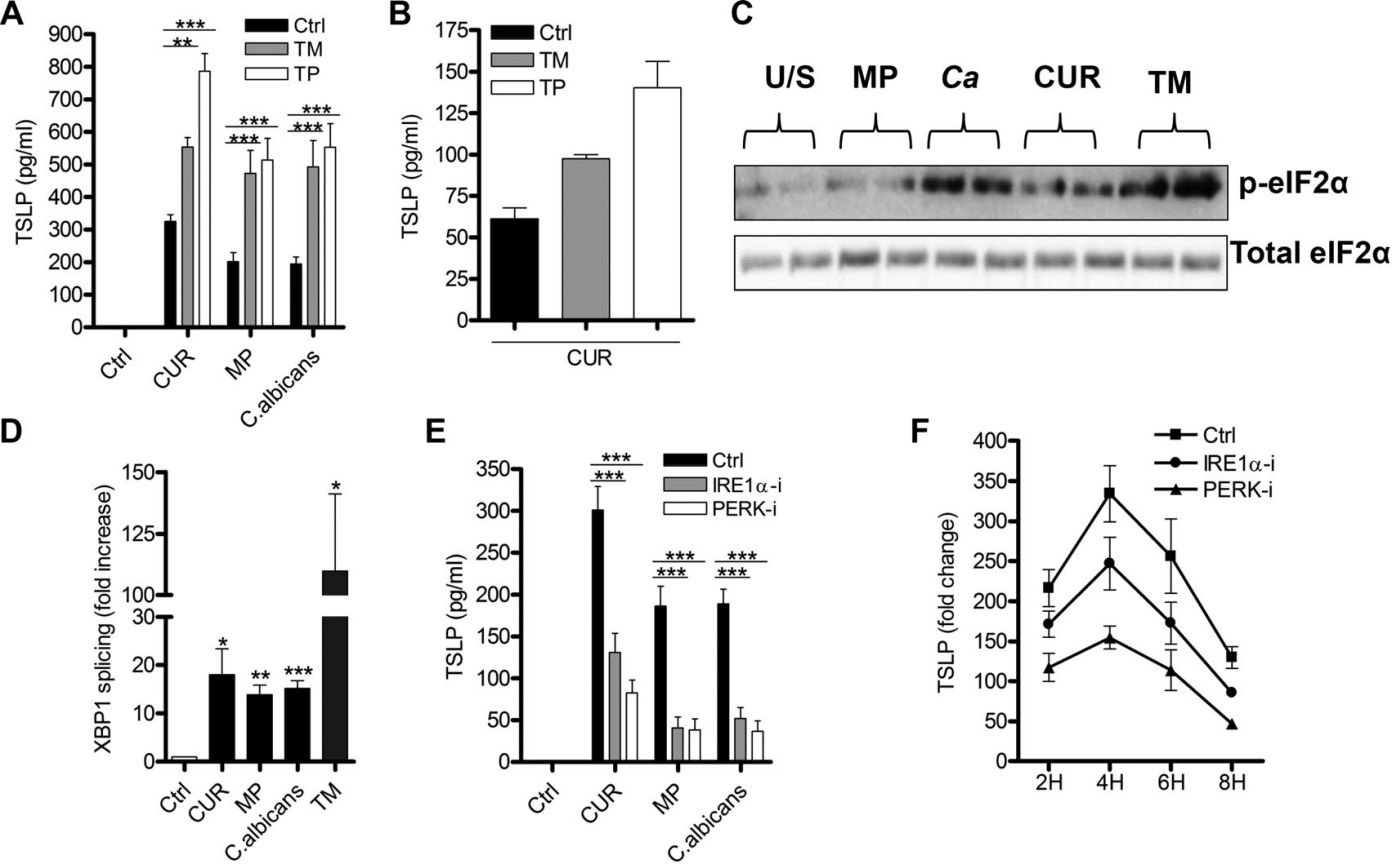
Dectin‐1 induces IRE1α and PERK and regulates TSLP production. (A) Human mDCs (*n* = 7 independent donors, presented as pooled data) or (B) human ex vivo CD1c^+^ DCs (*n* = 2 independent donors, presented as pooled data) were stimulated with CUR, MP, or *C. albicans* with or without the ER stress inducers tunicamycin (TM) or thapsigargin (TP) for 24 h. TSLP secretion was measured in 24‐hcell culture supernatants by ELISA. (C, D) Human mDCs were stimulated with CUR, MP, *C. albicans* (*Ca*) or TM for 4 h and analyzed for (C) phospho eIF2α (*n* = 2 independent donors represented) by immunoblot and (D) XBP‐1 splicing (*n* = 5 independent donors, presented as pooled data) by qRT‐PCR. (E, F) Human mDCs were preincubated for 1 h with or without IRE1α or PERK inhibitors and were then stimulated with (E) CUR, MP, or *C. albicans* for 24 h for TSLP secretion (*n* = 13 independent donors, presented as pooled data), measured by ELISA, or (F) 2, 4, 6, or 8 h for TSLP mRNA expression (*n* = 3 independent donors, data representative of single experiment performed in triplicate) by qRT‐PCR. Cumulative data are displayed as mean ± SEM. (D) Statistical significance calculated using *t* test where *p* values were calculated as unstimulated versus stimulated mDCs, XBP1 splicing *p* values for (CUR *p = 0.0339, (MP **p = 0.0029), (*C. albicans* ***p = 0.0010) and (TM *p = 0.0254). (A and E) Statistical significance was calculated using two‐way ANOVA with Bonferroni post‐tests (***p = 0.001, **p = 0.01).

Chemical induction of ER stress using tunicamycin (TM) or thapsigargin in conjunction with dectin‐1 stimulation increased TSLP secretion from mDCs (Fig. 3A), and from ex vivo CD1c^+^ DC (Fig. 3B). Importantly, mDCs subjected to ER stress in the absence of dectin‐1 stimulation were unable to induce TSLP (Fig. 3A), showing that ER stress modulates but is not sufficient to induce TSLP secretion, and further signifies that multiple independent signaling events are required.

ER stress results in the activation of three key sentinel proteins, IRE1α, PERK, and activating transcription factor 6 14, 26, 27, 28, 30. Activation of IRE1α induces RNAase activity leading to splicing of X‐box binding protein 1 (XBP1) mRNA to allow in‐frame translation and production of XBP1 protein, while activation of the kinase PERK results in eIF2α phosphorylation and activation of the integrated stress response (ISR) 14, 26, 27, 28, 30. The ability of dectin‐1 agonists to stimulate both eIF2α phosphorylation (Fig. 3C) and XBP1 splicing (Fig. 3D) suggests stimulation of the dectin‐1 pathway can activate components of the ER stress response. Although siRNA approaches were able to knockdown IRE1α and PERK mRNA expression, the degree of knockdown was not sufficient in DC to impact on downstream signaling pathways. However, we can conclude that IRE1α and PERK activation with dectin‐1 agonists did contribute important signals for TSLP expression, since specific inhibitors reduced dectin‐1 agonist‐induced TSLP from mDCs (Fig. 3E). The efficacy of the inhibitors was shown by loss of β‐glucan‐induced XBP1 splicing (Supporting Information Fig. 5A) and eIF2α phosphorylation (Supporting Information Fig. 5B).

The ISR can also be activated by other eIF2α kinases including protein kinase R (PKR). Although we show that β‐glucan stimulation potently activated PKR (Supporting Information Fig. 5C), inhibition of PKR had no effect on β‐glucan‐induced TSLP (Supporting Information Fig. 5D). This suggests that activation of PERK is critical for TSLP induction.

The ability of IRE1α or PERK inhibitors to reduce TSLP secretion suggests that ER stress signals were regulating TSLP expression at the level of gene transcription. Both IRE1α and PERK inhibition reduced TSLP mRNA expression during dectin‐1 stimulation without altering its kinetics of expression (Fig. 3F). In contrast, IRE1α inhibitor did not affect osteoprotegerin (OPG) mRNA expression and the PERK inhibitor had no effect on IL‐1β mRNA expression (Supporting Information Fig. 5E and F). The inability of ER stress inhibitors to modulate surface markers, CD83, CD86, and HLA‐DR, indicates that these inhibitors did not affect the maturation of mDCs induced by dectin‐1 agonists (Supporting Information Fig. 5G).

### TSLP secretion requires independent activation of the ER stress response and IL‐1R signaling

**Figure 4:**
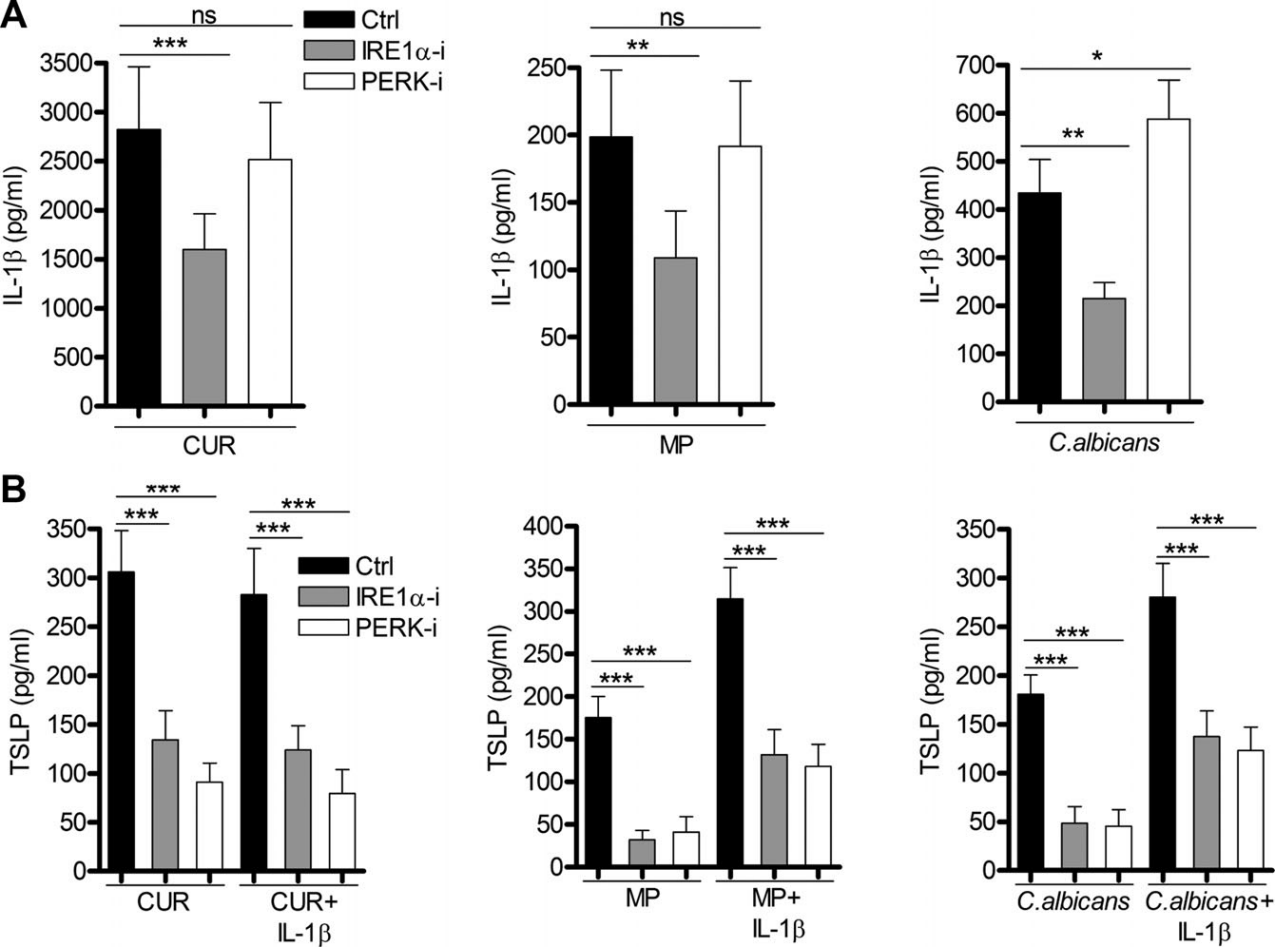
TSLP secretion requires independent activation of ER stress and IL‐1‐dependent signaling. (A) Human mDCs were preincubated for 1 h with or without IRE1α or PERK inhibitors and were then stimulated with CUR, MP, or *C. albicans* for 24 h (*n* = 12 independent donors, presented as pooled data). IL‐1β was measured in 24‐h cell culture supernatants by ELISA. (B) Human mDCs were preincubated for 1 h with or without IRE1α or PERK inhibitors and were then stimulated with CUR, MP, or *C. albicans* with or without recombinant IL‐1β for 24 h (*n* = 9 independent donors, presented as pooled data). TSLP was measured in 24‐h cell culture supernatants by ELISA. Cumulative data are displayed as mean ±SEM. Statistical significance was calculated using one‐way ANOVA with Bonferroni post‐tests (***p = 0.001, **p = 0.01, *p = 0.05).

The ability of ER stress to enhance TSLP expression raises the question whether IRE1α‐ and PERK‐regulated signals exhibit their effect indirectly by modulation of IL‐1β expression. Inhibition of IRE1α but not PERK activation reduced IL‐1β secretion from mDCs (Fig. 4A). The reduction of TSLP expression in the presence of the IRE1α inhibitor cannot be solely attributed to the loss of IL‐1β, since addition of exogenous recombinant IL‐1β did not rescue TSLP secretion (Fig. 4B). These data established that dectin‐1‐induced TSLP required independent signals from both the IL‐1 receptor and UPR‐stimulated sensors.

### The absence of CHOP does not affect dectin‐1‐induced TSLP expression by mDCs

Transcription factors induced during the UPR may be involved in the induction of TSLP gene expression. We have previously shown that ISR‐induced C/EBP homologous protein (CHOP) is a critical transcription factor in IL‐23p19 expression 14 and hypothesized a role in TSLP expression. We were able to reproduce this requirement for CHOP in the expression of IL‐23p19 (Supporting Information Fig. 6A) induced by β‐glucans in BMDCs, but found no role for CHOP in dectin‐1 activation of TSLP expression (Supporting Information Fig. 6B).

## Discussion

DCs are professional antigen‐presenting cells and have the ability to sense infection and danger signals and instruct immune cells to differentiate into different cell subsets. TSLP is an important cytokine in immune regulation 2, 4, 5, 6, 7, 8, 9, 10 and we have previously demonstrated that TSLP is expressed by human mDCs 14. In this study, we have shown that TSLP secretion from mDCs in response to stimulation with *C. albicans* or β‐glucans is regulated by an integration of signals from dectin‐1, the ER stress response, and the IL‐1 receptor.

We noted that mDCs generated significant levels of TSLP/cell (∼200–300 pg/mL from 0.5 × 10^5^ cells). It is difficult to directly compare TSLP production by mDCs and epithelial cells/fibroblasts; however, stimulation of these cell types typically generates ∼50–300 pg/mL from ∼2–4 × 10^5^ cells 8, 39. Furthermore, bronchoalveolar lavage fluids from severe asthmatic and chronic obstructive pulmonary disease patients have been shown to contain ∼50 pg/mL TSLP 40. Previous work in mice suggests that CD11c^+^ DC upregulate TSLP mRNA to a higher level compared to epithelial cells when both cell types are challenged with a stimulus to which both can respond 15, implying that mDCs may be more potent TSLP producers. Furthermore, contrary to a recent study showing that murine BMDCs do not secrete TSLP protein following stimulation with LPS and IL‐4 41, we have shown that BMDCs are capable of producing TSLP protein following stimulation with *C. albicans* and β‐glucan, though they may produce lower amounts than their human counterparts. It is likely that TSLP secretion is highly stimulation specific. Therefore, mDCs should not be discounted as potentially important sources of TSLP and we suggest this TSLP could have an important function during an initial fungal challenge.

Previous published work has shown that epithelial and stromal cells can be induced to express TSLP by the addition of exogenous cytokines such as IL‐1β and TNF‐α 8, 33, 35, 37, 38. In contrast, we showed that human mDCs had more stringent requirements for TSLP secretion and needed priming signals with a PRR agonist, such as β‐glucan, to induce significant TSLP production. These data suggest that dectin‐1 stimulation may allow modification of the TSLP promoter in immature mDCs that allows subsequent access to cytokine‐induced transcription factors.

Initially, we showed that dectin‐1‐induced TSLP from mDCs requires the activation of Syk, NF‐κB, and p38 MAPK, suggesting that promoter occupation by NF‐κB and signaling by p38 MAPK are requirements for TSLP induction. Furthermore, utilizing specific inhibitors of IL‐1β and TNF‐α, we identified that IL‐1β and not TNF‐α is critical for the TSLP induction pathway. A recent study using a skin irritation model for TSLP induction also identified IL‐1β as an important factor in the induction of TSLP in humans 37. Given that IL‐1β‐ and TNF‐α‐mediated signaling pathways induce some common downstream pathways including the activation of NF‐κB, it was surprising that TSLP induction from mDCs specifically required IL‐1 receptor signaling. These data suggest that there are qualitative differences in the downstream pathways induced by these cytokines. Further investigations into the unique factor/s stimulated by IL‐1 receptor signaling required for TSLP induction in human mDCs would be a worthwhile avenue of research.

We have previously shown that ER stress can increase TSLP mRNA expression 14 and hypothesized that the induction of the ER stress signaling components IRE1α and PERK via dectin‐1 engagement might contribute to TSLP production from mDCs. Utilizing specific inhibitors of the IRE1α‐ and PERK‐controlled UPR we were able to show that dectin‐1‐induced TSLP requires independent signals emanating from both IRE1α and PERK and the IL‐1 receptor.

These data suggest that dectin‐1 induction of ER stress modulates transcriptional regulation of TSLP via an IRE1α‐mediated arm. Therefore, the transcription factor XBP1, downstream of IRE1α activation, may be an important factor regulating TSLP expression in mDCs. We also suggest that PERK‐mediated eIF2α phosphorylation is a critical signal for the expression of TSLP. Although eIF2α phosphorylation can attenuate protein translation, it is apparent that activation of this pathway by dectin‐1 agonists is not sufficient to prevent the expression of TSLP protein. eIF2α phosphorylation can result in a transcriptional reprogramming of the cell via activation of the ISR 42. Transcription factors induced by the PERK‐controlled ISR may be involved in the induction of TSLP gene expression. We have previously shown that ISR‐induced CHOP is a critical transcription factor in IL‐23p19 expression 14 but we found no role for CHOP in β‐glucan activation of TSLP expression in murine BMDCs. Although we have found no putative ER stress associated transcription factor binding sites in the human and mouse TSLP promoter, gene expression is clearly ER stress responsive, therefore, a search for potential transcription factors mediating this effect would be worthwhile.

Whether diseases in which ER stress is known to be operating are associated with high levels of TSLP should be investigated, together with examination of any role for TSLP production in the manifestations of these diseases. Where TSLP is implicated in immunopathology, regulating the ER stress response could be useful therapeutically to decrease TSLP production in vivo.

TSLP is an established T_H_2‐cell differentiation factor, thus it is curious why it would be secreted in such high quantities during dectin‐1 stimulation. There is conflicting evidence on whether this signaling pathway induces or actively suppresses T_H_2‐cell‐associated cytokines and this appears to be very context dependent. Recent reports show that curdlan can inhibit T_H_2‐cell responses, promoting breast cancer progression 43; it was also reported to suppress IL‐5, IL‐13 and surprisingly IL‐1β from lymph nodes during epicutaneous sensitization 44. Conversely, β‐glucan treatment can increase T_H_2‐cell cytokine secretion from cocultured macrophages and lymphocytes 45 and stimulate production of both proallergic (IL‐33, CCL17, CCL22) and inflammatory (IL‐1β and CXCL1) mediators during lung exposure to *Aspergillus fumigatus*, in a dectin‐1‐dependent manner 46. Furthermore, in addition to TSLP we have observed that β‐glucan‐stimulated mDCs generate large quantities of CCL22 (data not shown) emphasizing that mDCs can secrete pro‐T_H_2‐cell factors.

What is the functional relevance of mDC‐derived TSLP? We suggest that mDCs are likely to be the major source of TSLP at nonepithelial sites, such as draining lymph nodes, and that this TSLP will have the potential to modulate the adaptive immune responses occurring there. This seems feasible, considering that Spadoni et al. have clearly established a functional role for DC‐derived TSLP, by its actions directly modulating T‐cell phenotype 16.

We have shown clear differences in the quantities of IL‐1β induced by different dectin‐1 agonists, whereas TSLP production remained stable. Pioneering experiments that established TSLP as a critical T_H_2‐cell driver administered large (15 ng/mL) quantities of this cytokine 3. This raises the possibility that the context of TSLP secretion in vivo may have significant effects on its ability to drive the differentiation of T_H_2/regulatory T cells or modulate the differentiation of T_H_17 cells 16, 22, 23, 24.

In summary, we have demonstrated that dectin‐1 stimulation of mDCs by *C. albicans* or β‐glucans produce TSLP and that this process involves the integration of signals from dectin‐1, the ER stress associated proteins IRE1α and PERK, and the IL‐1 receptor. These data argue that these signaling pathways may provide novel therapeutic targets to promote or downregulate TSLP production in relevant clinical settings.

## Materials and methods

### Cell isolation and generation of dendritic cells

CD14^+^ monocytes were purified from human PBMC by magnetic bead separation (Miltenyi) and were differentiated into mDCs with 20 ng/mL GM‐CSF (Life Technologies) and 4 ng/mL IL‐4 (BD Biosciences) by culturing for 6 days in RPMI1640 5% FCS as described previously 14. Ex vivo CD1c^+^ DC were isolated from human PBMC by magnetic bead separation (Miltenyi). Murine BMDCs were isolated from the femurs of wild‐type (WT) and CHOP KO C57B6 mice and cultured for 7 days in RPMI1640 10% FCS supplemented with 5% X63 conditioned media and 10 ng/mL IL‐4 (Peprotech).

### Cell stimulations

mDCs were seeded at a density of 2.5 × 10^5^/mL and were stimulated with 50 μg/mL of the β‐glucans, curdlan derived from *A. faecalis* (*A. faecalis*, Wako, Richmond, VA, USA), β‐1,3 glucan MPs derived from *S. cerevisiae* (David. L. Williams, East Tennessee State University) or heat‐killed hyphal *C. albicans* (MOI 2:1, gift from John Trowsdale, University of Cambridge) grown in sabouraud dextrose broth for 8 h at 37°C with shaking to an optical density of 0.2. *Candida albicans* was killed by heating for 1 h at 70°C.

### Reagents

Ten micrograms per millimeter mouse IgG_2B_ dectin‐1‐blocking antibody (clone‐259931 R&D Systems), 10 μg/mL mouse IgG_2B_ isotype control (clone‐20116 R&D Systems), 5 μM Syk inhibitor, R406 (Selleckchem), 10 μM NF‐κB inhibitor, isohelenin (Calbiochem), 1 μM p38 MAPK inhibitor, BIRB0796 (Selleckchem), 5 μg/mL anti‐TNF‐α, Infliximab (Jannsen Biotech), 1 μg/mL IL‐1 receptor antagonist (IL‐1RA; R&D Systems), 50 μM caspase‐1 inhibitor (Z‐YVAD‐FMK) (Calbiochem), 50 μM caspase‐8 inhibitor (Z‐IE(OMe)TD(OMe)‐FMK) (Calbiochem, 10 ng/mL TNF‐α (Biolegend), 10 ng/mL IL‐1β (Miltenyi), 1 μg/mL TM (Sigma‐Aldrich), 0.25 μM thapsigargin (Sigma‐Aldrich), 30 μM IRE1α inhibitor, 4μ8C (Calbiochem), and 1 μM GSK‐PERK inhibitor (Toronto Research). Where inhibitors, blocking antibodies and modifiers were used, mDCs were pretreated 1 h prior to cell stimulation. Repeated experiments were performed on independent donors unless otherwise stated.

### Cytokine production

mDC culture supernatants were harvested after 24 h of stimulation. The concentrations of TSLP (R&D Systems) and IL‐1β (eBioscience) were measured by ELISA as per the manufacturer's instructions.

### Quantitative real‐time PCR

Total RNA was extracted from cells using RNA Mini Kits (Bioline). Quantitative real‐time PCR was carried out using TaqMan Gene Expression Assays (Applied Biosystems) for TSLP, IL‐1β, and OPG and the SYBR Green method for XBP1 splicing. TSLP (Hs00263639_m1), IL‐1β (Hs00174097_m1), and OPG (Hs00171068_m1) TaqMan PCR probe primer sets (Applied Biosystems), and HPRT (MWG‐Biotech), probe, 5′‐TTCCTCATGGACTAATTATGGACAGG‐3′, forward, 5′‐TGAGGATTTGGAAAGGGTGTTT‐3′ reverse, 5′‐ACATCTCGAGCAAGACGTTCAG‐3′. SYBR primer sets (MWG‐Biotech), XBP1 spliced, forward 5′‐TGCTGAGTCCGCAGCAGGTG‐3′, reverse 5′‐GCTGGCAGGCTCTGGGGAAG‐3′, HGPRT, forward 5′‐GACACTGGCAAAACAATG‐3′, reverse 5′‐ACAAAGTCTGGCTTATATCC‐3′. Gene expression was normalized to HPRT and calculated as fold change from unstimulated control.

### Immunoblot

mDCs cell were lysed on ice in 250 μL of ice cold cytoplasmic lysis buffer (10 mM HEPES, 50 mM NaCl, 0.5 M Sucrose, 0.1 mM EDTA, 0.5% v/v Triton X‐100, 10 mM tetrasodium pyrophosphate, 17.5 mM β‐glycerophosphate, and one complete mini protease inhibitor cocktail tablet). After lysis the cytoplasmic extract was frozen at −20ºC overnight before thawing to aid cell lysis. Lysates were then centrifuged for 15 min at 4°C and the supernatant retained. Bradford assay was carried out to determine protein quantification (Thermo) and proteins were separated by SDS‐PAGE. Resolved proteins were detected after transfer by immunoblot with indicated primary antibodies, followed by incubation in HRP‐conjugated secondary antibodies and ECL (PerkinElmer) and visualized using GBox (Syngene). Monoclonal antibodies for phospho eIF2α (S51) (Abcam, 1090), eIF2α (Cell Signalling, 9722), phospho PKR (Thr451, Millipore, 07–886), PKR (Santa Cruz, sc‐798), IκBα (Santa Cruz, sc‐847), phospho Syk (Tyr525/525) (Cell Signalling, 2710), Syk (Cell Signalling, 13198), phospho p38 MAPK (Thr180 and 182) (Cell Signalling, 4511), p38 MAPK (Cell Signalling, 8690), and β‐actin (Abcam, 8226) were used for detection.

### Flow cytometry

mDCs were resuspended in ice cold PBS supplemented with 2 mM EDTA, 0.1% BSA, and 0.01% sodium azide, blocked with 10% mouse serum (Sigma) and stained with anti‐CD83 (BD), anti‐CD86 (BD), and anti‐HLA‐DR (Biolegend). Surface expression was evaluated relative to unstimulated stained controls on a BD CANTO II and analyzed using FlowJo software (Treestar).

### Data analysis

Data were analyzed using GraphPad Prism statistical package. Cumulative data are displayed as mean ± SEM. Statistical analysis using either *t* test (*p* values stated in figures legends) or one/two‐way ANOVAs with Bonferroni post‐tests *p < 0.05, **p < 0.01, ***p < 0.001.

## Conflict of interest

The authors declare no commercial or financial conflict of interest.

**Abbreviations**
BMDC: BM‐derived DC
CHOP: C/EBP homologous protein
eIF2α: eukaryotic initiation factor 2 α
HGPRT: Hypoxanthine‐guanine phosphoribosyltransferase
IRE1α: inositol‐requiring transmembrane kinase/endonuclease 1
ISR: integrated stress response
mDC: monocyte‐derived DC
MPs: microparticles
OPG: osteoprotegerin
PERK: protein kinase R‐like ER kinase
TSLP: thymic stromal lymphopoietin
UPR: unfolded protein response
XBP1: X‐box binding protein 1

## Supporting information

As a service to our authors and readers, this journal provides supporting information supplied by the authors. Such materials are peer reviewed and may be re‐organized for online delivery, but are not copy‐edited or typeset. Technical support issues arising from supporting information (other than missing files) should be addressed to the authors.

Peer review correspondence

Supporting Figures
