# Supplementary material for: TSLP production by dendritic cells is modulated by IL‐1β and components of the endoplasmic reticulum stress response
Source: Eur J Immunol. 2015 Dec 2;46(2):455–63. doi: 10.1002/eji.201545537 (PMC4783504; doi:10.1002/eji.201545537)
Supplement: Supplementary file 2 — Supporting Figures [file EJI-46-455-s002.pdf]

European Journal of Immunology

Supporting Information
for
DOI 10.1002/eji.201545537

Matthew J. Elder, Steven J. Webster, David L. Williams, J. S. Hill Gaston and Jane C. Goodall

TSLP production by dendritic cells is modulated by IL-1β and components of the endoplasmic reticulum stress response

© 2015 The Authors. *European Journal of Immunology* published by WILEY-VCH Verlag GmbH & Co. KGaA, Weinheim.

TSLP production by dendritic cells is modulated by IL-1β and components of the endoplasmic reticulum stress response

Supplementary Figures

Matthew J. Elder, Steven J. Webster,
David L. Williams, J. S. H. Gaston, Jane C. Goodall

Supplemental figure 1

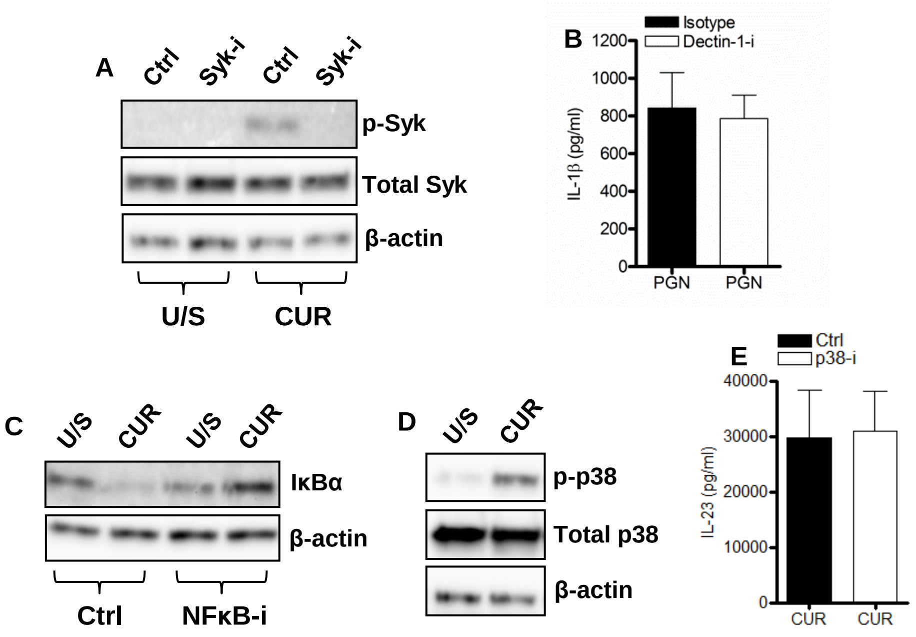

Supplemental Figure 1. β-glucan stimulated mDC induce Syk phosphorylation, IκBα degradation and p38 MAPK phosphorylation. (A) mDC were pre-incubated for one hour with or without the Syk inhibitor and were then stimulated with CUR for 4 hours. (B) mDC were pre-incubated for one hour with or without a dectin-1 neutralising antibody or isotype control and were then stimulated with peptidoglycan (PGN) for 24 hours (n=2). (C) mDC were pre-incubated for one hour with or without NFκB inhibitor and were then stimulated with CUR for 15 minutes and analysed for IκBα degradation. (D) mDC were stimulated with CUR for 4 hours and analysed for p38 MAPK phosphorylation. (E) mDC were pre-incubated for one hour with or without a p38 MAPK inhibitor and were then stimulated with CUR for 24 hours (n=3). IκBα degradation and Syk and p38 MAPK phosphorylation were measured by immunoblot. IL-1β and IL-23 were measured in 24 hour cell culture supernatants by ELISA. Cumulative data are shown as mean $\pm$SEM.

Supplemental figure 2

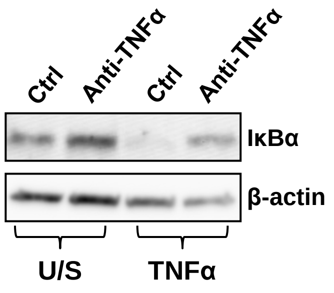

Supplemental Figure 2. Functionality of the anti-TNFα antibody utilised in this study is demonstrated by its ability to inhibit TNFα induced IκBα degradation. mDC were pre-incubated for one hour with or without anti-TNFα and were then stimulated with TNFα for 15 minutes. IκBα degradation was measured by immunoblot.

Supplemental figure 3

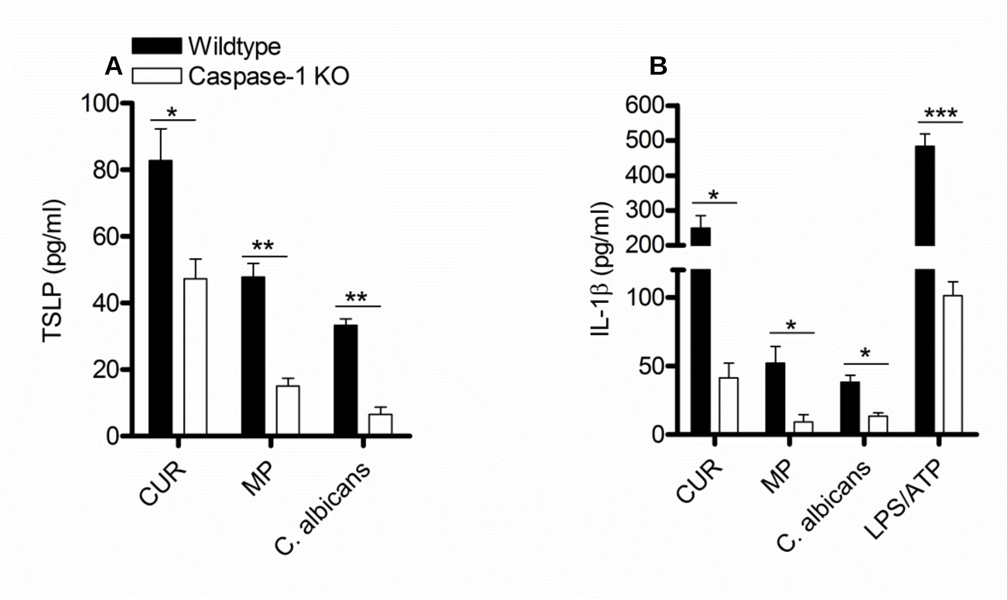

Supplemental Figure 3. Caspase-1 is required for dectin-1 induced TSLP and IL-1β. (A-B) BMDC from C57/B6 wild-type and caspase-1 KO mice were stimulated with CUR, MP, *C. albicans* or LPS/ATP for 24 hours (n=4). TSLP and IL-1β were measured in 24 hour cell culture supernatants by ELISA. Cumulative data are shown as mean ±SEM. Statistical significance was calculated using t test. p values for TSLP production by CUR stimulated BMDC (* p=0.0376), for MP stimulated BMDC (** p=0.0047) and for *C. albican*s stimulated BMDC (** p=0.0058). p values for IL-1β production by CUR stimulated BMDC (* p=0.0188), for MP stimulated BMDC (* p=0.0379), for *C. albican*s stimulated BMDC (* p=0.0253) and for LPS/ATP stimulated BMDC (*** p=0.0009).

Supplemental figure 4

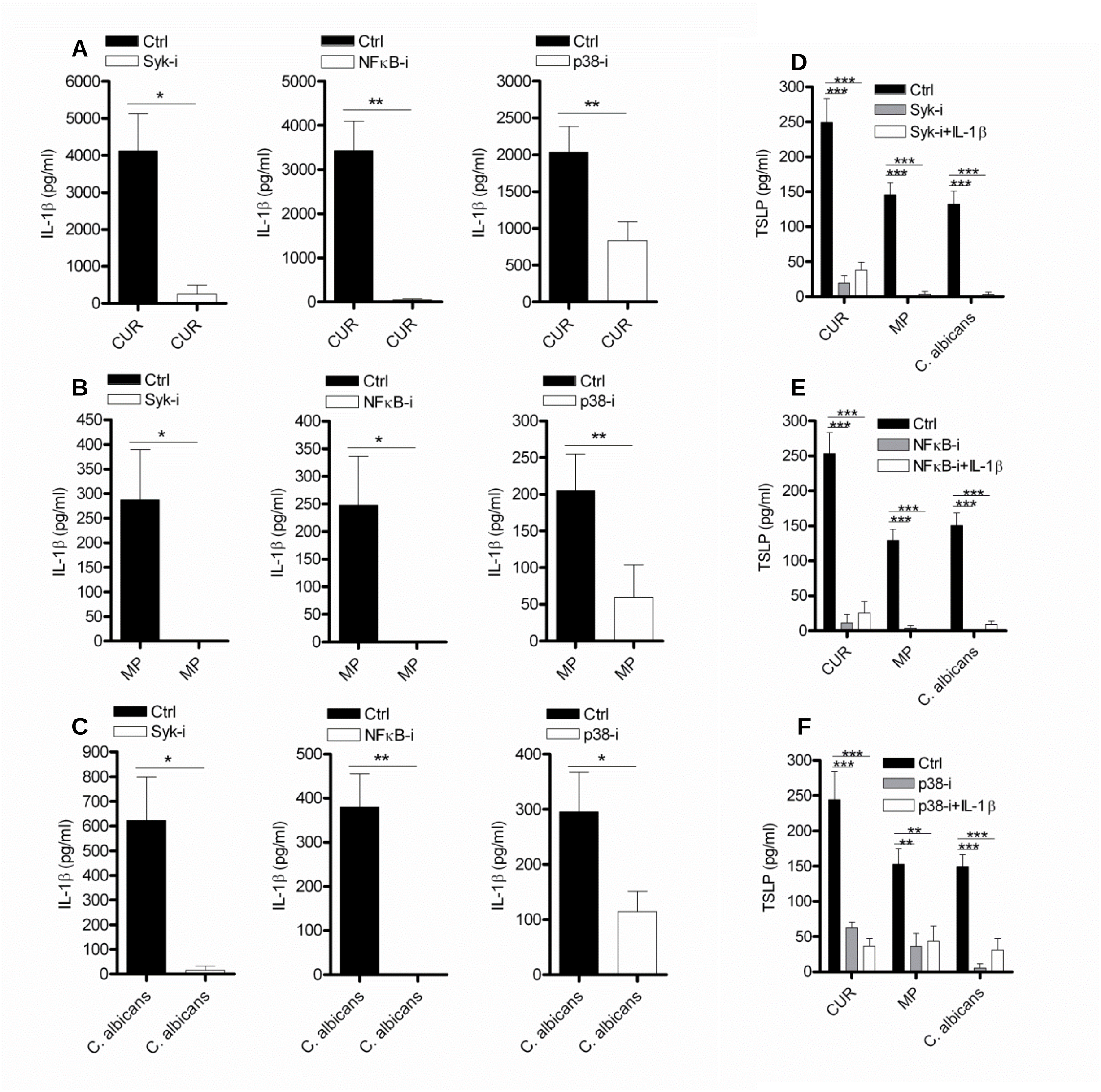

Supplemental Figure 4. Syk, NFκB and p38 MAPK are required for IL-1β and TSLP production. (A-C) mDC were pre-incubated for one hour with or without Syk inhibitor (n=5), NFκB inhibitor (n=5) or p38 MAPK inhibitor (n=7) and were then stimulated with CUR, MP or *C. albicans* for 24 hours. (D-F) mDC were pre-incubated for one hour with or without the Syk, NFκB or p38 MAPK inhibitors and were then stimulated with CUR, MP or *C. albicans* with or without recombinant IL-1β for 24 hours (n=3). TSLP and IL-1β were measured in 24 hour cell culture supernatants by ELISA. Cumulative data are shown as mean ±SEM. (A-C) Statistical significance calculated using t test. p values for IL-1β production by CUR stimulated mDC with Syk-i, NFκB-i and p38 MAPK-i respectively (* p=0.0127, ** p=0.0072 and ** p=0.0016), for MP stimulated mDC (* p=0.0491, * p=0.0488 and ** p=0.0036) and for *C. albicans* stimulated mDC (* p=0.0197, ** p=0.0073 and * p=0.0146). (D-F) Statistical significance was calculated using two-way ANOVA with Bonferroni post-tests. (*** p=0.001, ** p=0.01).

Supplemental figure 5

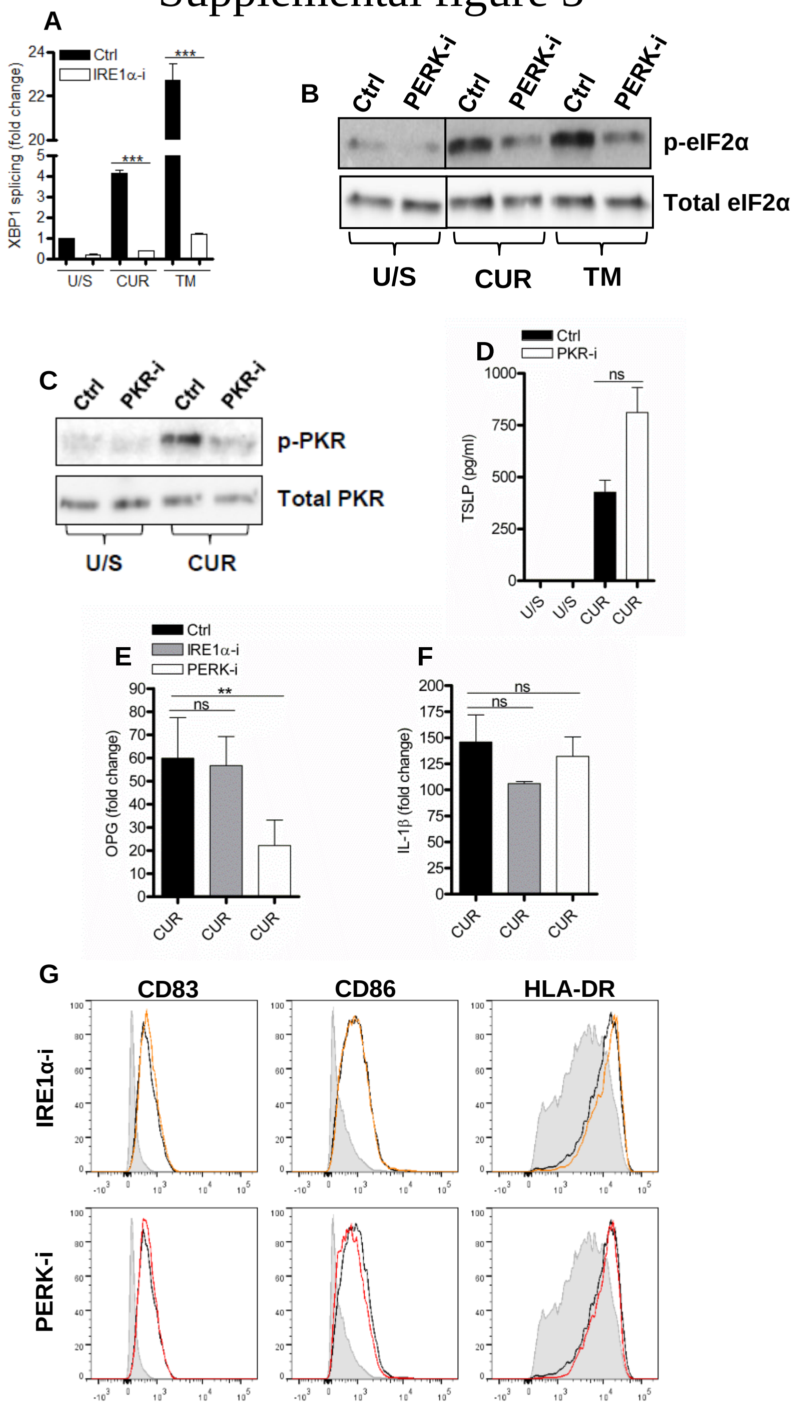

Supplemental Figure 5. Inhibitors of IRE1α and PERK inhibit XBP1 splicing and eIF2α phosphorylation respectively, but do not affect DC activation markers. (A-B) mDC were pre-incubated for one hour with or without IRE1α or PERK inhibitors and were then stimulated with CUR or TM for 4 hours, and analysed for XBP1 splicing (n=3, representative experiment shown) and phospho eIF2α (n=2, representative experiment shown) analysed as functional readouts for the efficacy of inhibitors. (C-D) mDC were pre-incubated for one hour with or without PKR inhibitor and were then stimulated with CUR for (C) 4 hours for PKR phosphorylation 4 hours or (D) 24 hours for TSLP secretion (n=2). (E-G) mDC were pre-incubated for 1 hour with or without IRE1α or PERK inhibitors and were then stimulated with CUR for 4 hours analysed for OPG and IL-1β mRNA expression and for CD83, CD86 and HLA-DR surface expression by flow cytometry. Unstimulated control (grey filled profile), CUR stimulated (black line profile), CUR plus IRE1α or PERK inhibitors (grey line profile). XBP1, OPG and IL-1β mRNA expression were measured by qRT-PCR. PKR phosphorylation was measured by immunoblot. TSLP was measured in 24 hour cell culture supernatants by ELISA. Cumulative data are shown as mean ±SEM. Statistical significance calculated using one-way ANOVA with Bonferroni post-tests (ns = not significant, *** p=0.001, ** p=0.01, * p=0.05).

Supplemental figure 6

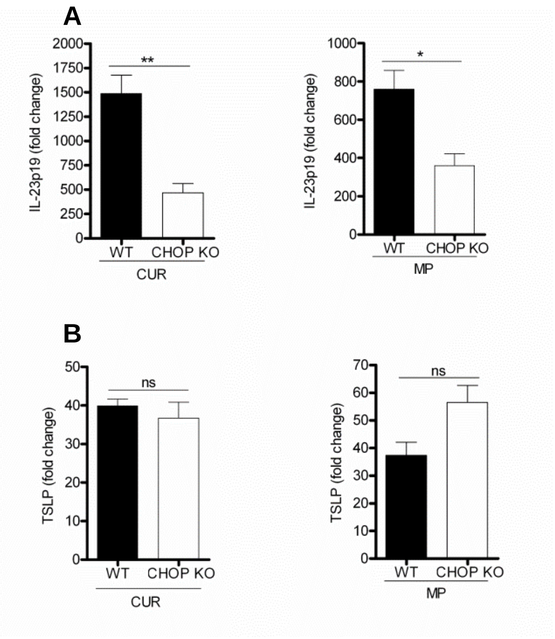

Supplemental Figure 6. The absence of CHOP does not affect dectin-1 induced TSLP expression. (A-B) BMDC from C57/B6 wild-type and CHOP KO mice were stimulated with CUR and MP for 4 hours (n=4). TSLP and IL-23p19 mRNA expression were measured by qRT-PCR. Cumulative data are shown as mean ±SEM. Statistical significance was calculated using t test. p values for IL-23p19 expression by CUR stimulated BMDC (** p=0.0029), and for MP stimulated BMDC (* p=0.0143).
