# Supplementary material for: TSLP production by dendritic cells is modulated by IL‐1β and components of the endoplasmic reticulum stress response
Source: Eur J Immunol. 2015 Dec 2;46(2):455–63. doi: 10.1002/eji.201545537 (PMC4783504; doi:10.1002/eji.201545537)
Supplement: Supplementary file 1 — Peer review correspondence [file EJI-46-455-s001.pdf]

# Peer review correspondence

European Journal of Immunology

**TSLP production by dendritic cells is modulated by IL-1β and components of the endoplasmic reticulum stress response**

Matthew J. Elder, Steven J. Webster, David L. Williams, J. S. Hill Gaston, Jane C. Goodall

Corresponding author: J. S. Hill Gaston, Department of Medicine, University of Cambridge, School of Clinical Medicine, Addenbrookes Hospital, Cambridge, United Kingdom

---



| | | |
|---|---|---|
| Review Timeline: | Submission date: | 30 January 2015 |
| | First editorial decision: | 25 February 2015 |
| | First revision received: | 5 May 2015 |
| | Second editorial decision: | 21 May 2015 |
| | Second revision received: | 2 October 2015 |
| | Accepted: | 9 November 2015 |



---

Handling Executive Committee members: Prof. Caetano Reis e Sousa

Please note that the correspondence below does not include the standard editorial instructions regarding preparation and submission of revised manuscripts, only the scientific revisions requested and addressed.

<u>First Editorial Decision – 25 February 2015</u>

Dear Mr. Elder,

Manuscript ID eji.201545537 entitled "TSLP production by dendritic cells is modulated by IL-1β and the endoplasmic reticulum stress response" which you submitted to the European Journal of Immunology has been reviewed. The comments of the referees are included at the bottom of this letter.

A revised version of your manuscript that takes into account the comments of the referees will be reconsidered for publication. You will see that referees 2 and 3 feel that the impact and novelty of your story could be enhanced by data outlining the molecular mechanism of how low UPR induction, driven by dectin, enhances TSLP production. We therefore strongly encourage you to fully address the concerns of referees 2 and 3 in your revised submission.

European Journal of Immunology

You should also pay close attention to the editorial comments included below. *In particular, please edit your figure legends to follow Journal standards as outlined in the editorial comments. Failure to do this will result in delays in the re-review process.*

Please note that submitting a revision of your manuscript does not guarantee eventual acceptance, and that your revision will be re-reviewed by the referees before a decision is rendered.

If the revision of the paper is expected to take more than three months, please inform the editorial office. Revisions taking longer than six months may be assessed by new referees to ensure the relevance and timeliness of the data.

Once again, thank you for submitting your manuscript to European Journal of Immunology and we look forward to receiving your revision.

Yours sincerely,
Karen Chu

On behalf of Prof. Caetano Reis e Sousa

Dr. Karen Chu
Editorial Office
European Journal of Immunology
e-mail: ejied@wiley.com
www.eji-journal.eu

***********************************

Reviewer: 1

Comments to the Author
In this study the authors investigate the signals that regulate the production and secretion of human TSLP by monocyte-derived DC (and blood CD1c plus DC) upon treatment with the dectin-1 agonist beta-glucans. The authors showed that peIF2alpha, and XBP1 splicing (two parkers of ER-stress) are induced by beta-glucans. They also showed that IL-1 is released upon treatment.
Using chemical/biological inhibitors of NFkB, P38, IL-1, IRE1 and PERK they demonstrate that these pathways co-operate to trigger the production of TSLP in mDCs, however the data suggest that the ER-stress pathway may affect TSLP1 mRNA expression rather than exclusively contributing to IL-1 production.

While the study mostly rely on inhibitors, the activity of the inhibitors (except for caspase-1/8) has been controlled and reported in supplementary data.

Suggestion:
In order to place the events in order of sequence, it would be interesting to complete the study by investigating the contribution of the p38/NFkB and Syk pathways to the IL-1 production (role in pro-IL1 expression or inflammasome priming/activation)

Reviewer: 2

Comments to the Author
In the manuscript entitled "TSLP production by dendritic cells is modulated by IL-1β and the endoplasmic reticulum stress response" the authors show that ligands of Dectin-1, a PRR involved in fungal recognition, are potent inducers of TSLP in human cultures of dendritic cells. Based on the use of blocking antibodies and chemical inhibitors, the authors state that Dectin-1 signals for the production of TSLP via two mechanisms that operate independently: one controlled by the cytokine IL-1β and another dependent on the sensors of the unfolded protein response IRE-1α and PERK. Although the mechanisms controlling the production of TSLP in human DC cultures are of interest for the scientific and clinical community, this reviewer considers that the current manuscript does not provide a clear understanding of the mechanisms regulating TSLP production. Overall, the interpretation of the data is complicated and the conclusions drawn are preliminary based on the evidence presented in the manuscript. On one hand, the work is largely based on the use of chemical inhibitors and blocking antibodies, some of which are shown in absence of their correspondent specificity and toxicity controls. In addition, this reviewer considers that the evidence supporting the ability of Dectin-1 to trigger endoplasmic reticulum stress is insufficient. Finally, there are discrepancies throughout the manuscript and with the literature that need to be addressed.

Major concerns are:
• The authors show that stimulation of monocyte derived DC with Curdlan or β-1,3 glucan microparticles (two types of Dectin-1 ligands) or heat killed C. albicans trigger the production of TSLP at similar levels.
This is very surprising, and at variance with the literature showing that β-1,3 glucan microparticles are markedly weaker than curdlan at stimulating DC due to their small size, which renders them prone to phagocytosis leading to attenuation of Dectin 1 signalling (Hernanz-Falcon et al 2009, EJI). The distinction between these two types of ligands in their ability to elicit Dectin-1-dependent responses and production of inflammatory cytokines has also been noticed in additional cell types including macrophages (Rosas et al 2008, JI; Goodridge et al 2011, Nature). With this body of evidence, how do the authors explain the potent efficacy of β-1,3 glucan microparticles in the production of TSLP in their system? Why is this specific to this cytokine?. Authors should reconcile their data with previous published work. Also they should explain

European Journal of Immunology

why in fig 2D and 2E β-1,3 glucan microparticles induce much lower levels of TSLP than curdlan. This major discrepancy needs to be resolved.

• The authors use a blocking antibody to Dectin-1 and observe a decrease in TSLP production in response to stimulation with Curdlan, β-1,3 glucan microparticles or heat killed C. albicans. Whereas this is an important finding, the experiment lacks the appropriate control, which should be an isotype-matched antibody and not media as described. In addition, specificity of the blockade should be addressed by measuring a Dectin-1-independent cytokine produced in response to C. albicans stimulation.

• The authors observe that the use of IL-1RA but not anti-TNFα antibody prevents TSLP production in response to stimulation with curdlan, β-1,3 glucan microparticles or heat killed C. albicans. The authors also show that these stimuli trigger IL-1β production in mDC, which is consistent with previous work. Nevertheless, the authors observe that in response to curdlan, processing of IL-1β depends on caspase 1 and caspase 8 as seen by the use of inhibitors. These data contrast to previous work (Gringhuis et al 2012, Nature Immunology) showing that curdlan does not induce activation of caspase 1 and that curdlan-dependent production of IL-1β in human mDC is caspase1-independent. Authors should reconcile the present data with the established findings and provide experimental evidence validating their reagents, as it is of relevance for the manuscript. In addition, the study would greatly benefit from experiments using RNA-mediated interference although this may be challenging when using human cultures of DCs.

• The authors show that chemical induction of the endoplasmic reticulum (ER) stress response synergizes with dectin-1 ligands to enhance TSLP production in agreement with previous work published by the same group. In this work, the authors measure the induction of XBP-1 spliced and phospho-eif2α as a readout of ER stress. In this regard, this reviewer feels that these two parameters are insufficient to claim that Dectin-1 signalling triggers a complete ER stress response. Several pathogens and TLR ligands are able to specifically activate IRE-1α and XBP-1s in absence of a canonical unfolded protein response (Martinon et al 2010, Nature Immunology). Also, considering that eif2α can be phosphorylated by additional members of the integrated stress response (PKR, GCN2, HRI and PERK), it is not a clear readout of ER stress induction. Authors should provide additional experimental evidence to solidify their findings. This evidence include a conventional PCR for both forms of XBP-1 (unspliced and spliced), induction of XBP-1, ATF6 and ATF4 target genes and western blots for phospho IRE-1α, phospho PERK, Bip and XBP-1s in response to Dectin-1 stimulation.

• The authors observe that inhibitors of IRE-1α and PERK prevent TSLP production. Although this is an interesting finding, is this specific for TSLP? it is needed to show a cytokine not regulated by the unfolded protein response to assess specificity of the pathway.

Additional remarks

The materials and methods section is poorly written and insufficient for a manuscript in European Journal of Immunology. The information provided does not allow a clear understanding of the experimental settings. For instance, no reference is made to the pre-treatment times, particularly in experiments using blocking antibodies, inhibitors or activators. These descriptions are extremely important for appropriate interpretation of the data. In addition, no reference is made to the details (such as catalogue number) of the blocking antibody to Dectin-1.

Reviewer: 3

Comments to the Author
This paper investigates the contribution of different signaling cascades to the production of thymic stromal lymphopoietin (TSLP) by dendritic cells (DC). Upon Dectin-1 stimulation, TSLP production by DC is greatly enhanced by IL-1b, but not TNFa, and is even further increase by signals emanating from the endoplasmic reticulum (ER) stress response. Dectin triggering seems to drive activation of both IRE-1 and PERK, leading to amplification of TSLP production. Although these are interesting observations, they are not unexpected and the authors mostly use pharmacological inhibitors and in vitro assays to reach to their conclusion. As this work also lacks a detailed characterization of the molecular machineries at work during this process, the manuscript would really improve in quality if the authors would document at the molecular level why the relatively low UPR induction driven by dectin enhances TSLP production and synergizes with IL-1b.

Main points.
1) Pharmacological inhibitors studies should be complemented by siRNA approaches. Levels of cell death induced by the inhibitors should be documented

2) Activation of the UPR by dectin stimulation should be documented better by determining XBP1 protein levels, impact of XBP1 targets (qPCR and protein), impact on potential RIDD targets (qPCR and protein). Indeed the level of XBP-1 mRNA splicing measured in Fig 3D are relatively low (12-18 %?) and further demonstration of XBP1 activity is required to strengthen the current observations. Next, it will be important to establish whether TSLP1 production increase results from augmenting XBP-1 expression or from an alternative implication of the RIDD activity of IRE-1. Is there an implication of the RIGI/IPS-1 pathway has suggested by Cho et al. 2013? siRNA approaches should help resolving this matter.

3) As for the PERK pathway activity, the phospho-blot presented here is not of great quality. Better quality data and kinetics of eIF2 phosphorylation should be shown over several hours, matching the time of TSLP production. Indeed phosphorylation of eIF2a has been documented for monocyte-derived DCs after LPS

stimulation with a different outcome that the one presented here for dectin. A comparative monitoring should be performed between dectin and TLR stimulated cells over time. Global protein synthesis intensity should also be monitored in parallel to TSLP and other cytokines transcription as well as their translation. Indeed NFk-B activation has been shown to be augmented upon translation reduction resulting in a lack of Ik-b neoynthesis (see fig 2). Phospho-PERK accumulation over time should also be documented and linked to dectin stimulation and ER stress. ATF4 expression and other P-eIF2a sensitive molecules should also be monitored.

4) No evaluation on IL-1b expression and its processing is provided in the current version of the manuscript. It will be important to establish the impact of Dectin 1 and UPR pathways on inflammasome activity and IL-1 processing.

5) An attempt should be made to have the demonstration that UPR is induced significantly upon Candida infection or dectin stimulation in DCs or macrophages in vivo, to give physiological relevance to the findings.

First revision – authors' response – 5 May 2015

Reviewer: 1

1. While the study mostly rely on inhibitors, the activity of the inhibitors (except for caspase-1/8) has been controlled and reported in supplementary data. The caspase 1/8 inhibitors which we used are well described in the literature, so we did not perform tests for non-specific toxicity; their efficacy is shown by their potent inhibition of IL-1 secretion as expected, Fig. 2F.

2. In order to place the events in order of sequence, it would be interesting to complete the study by investigating the contribution of the p38/NFkB and Syk pathways to the IL-1 production (role in pro-IL1 expression or inflammasome priming/activation)

In response to the reviewer's comment on contribution of Syk, p38 and NFkB to IL-1 secretion, we have evaluated this in the presence of Syk, p38 and NFkB inhibitors (supplementary Figure 4). As with TSLP, IL-1 production is reduced in the presence of these inhibitors. Furthermore as Syk, p38MAPkinase and NFkB inhibitors reduce IL-1 secretion, we stimulated mDC with dectin-1 agonists with or without added recombinant IL-1β following pre-incubation with Syk, p38 and NFkB inhibitors, to investigate whether Syk, p38MAPK and NFkB simply modulate TSLP secretion by their ability to reduce IL-1β expression. Our data shows that even when IL-1β is added, TSLP expression is still significantly reduced, suggesting that Syk, p38 and NFkB affect TSLP expression directly. The manuscript has been edited accordingly (supplemental figure 4 and lines 162-165).

Reviewer: 2

General points

The work is largely based on the use of chemical inhibitors and blocking antibodies, some of which are shown in absence of their correspondent specificity and toxicity controls.

In response to the reviewer's comment on the use of inhibitors, we have shown, in both the main figures and supplementary figures, efficacy of the inhibitors and their effects on TSLP secretion. We have now also included additional figures that further support the use and specificity of these reagents, as follows:

• Anti-Dectin-1 antibody – Inhibits dectin-1 agonist induced TSLP (figure 1D) but does not affect peptidoglycan induced IL-1β (supplemental figure 1B). The manuscript has been modified accordingly (lines 108-112)

• We show that Syk, NF-kb and p38MAPkinase are required for TSLP expression using well established chemical inhibitors. As mentioned above, to demonstrate that this reduction was not due to an indirect effect of reducing IL-1 secretion (which is essential for TSLP expression), we tested these inhibitors in the presence of added IL-1 and showed that TSLP expression was still significantly reduced (additional supplemental figure 4D-F). The manuscript has been modified accordingly, lines 162-165.

• We show that anti-TNF does not affect dectin-1 induced TSLP and provide an important control in supplementary data figure 2, showing that this antibody efficiently neutralises TNFa activity, as demonstrated by blockade of TNFa, mediated activation of NF-kB, supplementary data Figure 2). This data is important as TNFa has been previously shown to modulate TSLP production in epithelial cells.

• Dectin-1 induced TSLP is reduced in the presence of IRE1 and PERK inhibitors. To test whether these inhibitors affected cytokine production generally we evaluated expression of other cytokines. Thus the IRE1 inhibitor did not affect osteoprotegerin mRNA expression and the PERK inhibitor had no effect on IL-1 mRNA expression (supplemental figure 5E-F). The manuscript has been modified accordingly, lines 198-200. In addition, the IRE1 and PERK inhibitors had no effect on DC activation status (supplemental figure 5G).

Major concerns are:

1. The authors show that stimulation of monocyte derived DC with curdlan or β-1,3 glucan microparticles (two types of Dectin-1 ligands) or heat killed C. albicans trigger the production of TSLP at similar levels. This is very surprising, and at variance with the literature showing that β-1,3 glucan microparticles are markedly weaker than curdlan at stimulating DC due to their small size, which renders them prone to phagocytosis leading to attenuation of Dectin 1 signalling (Hernanz-Falcon et al 2009, EJI). The distinction between these two types of ligands in their ability to elicit Dectin-1-dependent responses and production of inflammatory cytokines has also been noticed in additional cell types including macrophages (Rosas et al 2008, JI; Goodridge et al 2011, Nature). With this body of evidence, how do the authors explain the potent

efficacy of β-1,3 glucan microparticles in the production of TSLP in their system? Why is this specific to this cytokine? Authors should reconcile their data with previous published work. Also they should explain why in fig 2D and 2E β-1,3 glucan microparticles induce much lower levels of TSLP than curdlan. This major discrepancy needs to be resolved.

We agree that this is a very important point, but we do not think that our work is at variance with previous studies and we explain below and have made changes to the manuscript to explain why. As a supporting note to this section, it is important to mention that we obtained our beta-1,3 microparticles from the Professor David L Williams, who also supplied the reagents used in the Hernanz-Falcon et al. publication. The microparticles have rigorous quality control procedures and are tested for endotoxin contamination.

We are in agreement with the literature showing that β-1,3 glucan microparticles are markedly weaker than curdlan at inducing certain DC-derived inflammatory cytokines including IL-6, IL-23 and IL-12. This data is in a manuscript in preparation, and we have discussed it on lines 136-138 in the manuscript. It is important to emphasize that Hernanz-Falcon et al 2009 and Rosas et al 2008, did not examine TSLP expression (nor IL-1) in their studies. We show that the large glucan particles (curdlan) induce much more IL-1, than do glucan microparticles, but importantly,we show that the small quantity of IL-1 induced by glucan microparticles is biologically significant for TSLP expression. This is demonstrated by the clear loss of TSLP expression induced by glucan microparticles when IL-1 signalling is inhibited (Figure2B). This suggests that there is a low threshold for IL-1 signalling to enable TSLP expression and that the small quantity of IL-1 induced by glucan microparticles, is sufficient in combination with dectin 1 signals to induce comparable quantities of TSLP; this may explain why TLSP expression is relatively insensitive to particle size.

Reviewer 2 has highlighted the differences in the TSLP secreted in response to curdlan and microparticles in figure2D and 2E, as compared to figure 2B. The data in figure 2B show no significant differences in TSLP secretion and represents an independent cohort of donors from figure 2D and 2E. It is important to highlight that we also showed identical TSLP secretion induced by different size particles using murine DCs. With regard to the differences shown in other figures, please note that the time course of TSLP and IL-1 □secretion in response□to curdlan and microparticles in Fig 2D represents data from a single donor (hence the small error bars, which are experimental replicates) and here individual donor variation is evident (as would be expected). Where we do show differences, the amounts of TSLP produced only varied over a 2-fold range, whether using curdlan or microparticles. In contrast IL-1 (and IL-6/IL-23, data not shown) showed a 100 -fold difference between curdlan and microparticles. Microparticle induced TSLP can also be boosted a small amount by the addition of recombinant IL-1 □□although IL-1β alone is not sufficient to induce TSLP. Thus the somewhat lower amount of TSLP secretion induced by microparticles in Figure 2D/E may be due to the lower quantity of IL-1 they induce. In agreement with this interpretation, addition of recombinant IL-1 resulted in identical TSLP expression induced by curdlan or

European Journal of Immunology

microparticles (figure 2C), again confirming that the critical signals for TSLP gene that are not dependent on IL-1 signals are independent of particle size.

We also found that the chemokine, CCL22 (which is dectin 1 dependent, data not shown) is secreted at equivalent quantities by mDC whether stimulated with curdlan or glucan microparticles (manuscript lines, 298-9). This provides a further example where glucan particle size does not influence cytokine output by dendritic cells.

Indeed, we suggest in a publication in preparation, that the strength of activation of the inflammasome may be the critical component that distinguishes the pro-inflammatory cytokine production (IL-6 and IL-23) by different particle sizes. This is demonstrated by the ability of exogenous IL-1 to restore IL-6 and IL-23 secretion induced by microparticles to equivalent levels detected when stimulating with curdlan (data not shown, manuscript in preparation).

2. The authors use a blocking antibody to Dectin-1 and observe a decrease in TSLP production in response to stimulation with Curdlan, β-1,3 glucan microparticles or heat killed C. albicans. Whereas this is an important finding, the experiment lacks the appropriate control, which should be an isotype-matched antibody and not media as described. In addition, specificity of the blockade should be addressed by measuring a Dectin-1-independent cytokine produced in response to C. albicans stimulation.

In response to the reviewer's comment that the dectin-1 blocking experiments were not properly controlled, we apologise that figure 1D was mislabelled and this has been amended to indicate that an isotype-matched control antibody was used.

In response to the reviewer's comment suggesting we should find a dectin-1 independent cytokine response to C. albicans stimulation, we have tested a large panel of cytokines in response to C. albicans in the presence of the dectin-1 neutralisation antibody and found dectin-1 to be essential for all these responses (data not shown). However, we have stimulated human mDC with peptidoglycan (TLR2/NOD2 activator) and shown IL-1 secretion to be independent of dectin-1 signalling. This clearly shows that the dectin 1 antibody does not block cytokine responses non-specifically (supplemental figure 1B and manuscript lines 111-112).

3. The authors observe that the use of IL-1RA but not anti-TNFα antibody prevents TSLP production in response to stimulation with curdlan, β-1,3 glucan microparticles or heat killed C. albicans. The authors also show that these stimuli trigger IL-1β production in mDC, which is consistent with previous work. Nevertheless, the authors observe that in response to curdlan, processing of IL-1β depends on caspase 1 and caspase 8 as seen by the use of inhibitors. These data contrast to previous work (Gringhuis et al 2012, Nature Immunology) showing that curdlan does not induce activation of caspase 1 and that curdlan-

European Journal of Immunology

dependent production of IL-1β in human mDC is caspase1-independent. Authors should reconcile the present data with the established findings and provide experimental evidence validating their reagents, as it is of relevance for the manuscript.

We are in agreement with Gringhuis et al that caspase-8 is essential for dectin-1 induced IL-1; however, we also observe a crucial role for caspase-1 (Figure 2F) which, as the reviewer highlights, is in disagreement with this publication. Technically, we have used the same approach and identical caspase-1/8 inhibitors, at the same concentration as Gringhuis et al. It is worth noting that human dendritic cells in our hands produce 10-fold more IL-1 than reported by Gringhuis et al and this may have enhanced our ability to detect an effect of caspase 1 inhibition. To provide further support to our findings, we have obtained additional evidence using dendritic cells derived from mice with a targeted deletion of caspase 1 and wild-type controls. These data clearly show that caspase-1 is required for β-glucan induced IL-1 and TSLP in murine DCs (supplemental figure 3A-B, and reference in the manuscript on lines 157-159).

Most importantly, our finding is not at odds with other publications which have shown an essential role for caspase-1 in dectin-1 induced IL-1β (Gross et al Nature 2009. 459: 433-436.) and more recently by Ganesan et al (J Immunol 2014. 193: 2519-2530).

In addition, the study would greatly benefit from experiments using RNA-mediated interference although this may be challenging when using human cultures of DCs.

We agree that experiments with RNAi would provide a very useful approach to address our questions, but as the reviewer states, RNA interference is technically difficult in primary DC. We have extensive experience in RNA-mediated interference in macrophage/DC like cell lines (THP-1 and U937); however, since these cell lines do not produce TSLP, they are not suitable for this study.

5. The authors show that chemical induction of the endoplasmic reticulum (ER) stress response synergizes with dectin-1 ligands to enhance TSLP production in agreement with previous work published by the same group. In this work, the authors measure the induction of XBP-1 spliced and phospho-eIF2α as a readout of ER stress. In this regard, this reviewer feels that these two parameters are insufficient to claim that Dectin-1 signalling triggers a complete ER stress response. Several pathogens and TLR ligands are able to specifically activate IRE-1α and XBP-1s in absence of a canonical unfolded protein response (Martinon et al. 2010, Nature Immunology).

We agree with reviewer 2 that we do not provide data to delineate the complete UPR induced by dectin-1 signalling. Instead, we clearly show that dectin-1 activation can induce both XBP1 splicing and eIF2a phosphorylation, which are indicators of the activation of proximal ER stress sensors, (figure 3C & D). Using specific inhibitors, we establish that dectin-1 induced XBP1 splicing is dependent on IRE1, and that

European Journal of Immunology

eIF2a phosphorylation requires PERK activation (supplementary figure 5A-B). Additionally we have used tunicamycin as a positive control for ER stress induction and show the specific inhibitors inhibit tunicamycin induced XBP1 and eIF2a phosphorylation in mDC. The aim of the paper is to show that canonical components of the UPR (IRE1 and PERK) are activated by dectin-1 signalling, and that this activation is required for TSLP secretion, rather than to describe in detail the UPR induced by dectin-1 signalling.

As the reviewer noted, it has previously been shown that activation of IRE1 by LPS makes an important contribution to expression of IL-6 and TNFa, yet does not activate an identical UPR signature to that detected by potent chemical inducers of ER stress (Martinon et al, 2013 Nature Immunology); we have similar data for LPS responses (manuscript in preparation). We have modified the manuscript to indicate that our emphasis was to detect activation of proximal ER stress sensors in TSLP expression but not to delineate in full the dectin-1 induced UPR.

4. Considering that eIF2α can be phosphorylated by additional members of the integrated stress response (PKR, GCN2, HRI and PERK), it is not a clear readout of ER stress induction. Authors should provide additional experimental evidence to solidify their findings. This evidence include a conventional PCR for both forms of XBP-1 (unspliced and spliced), induction of XBP-1, ATF6 and ATF4 target genes and western blots for phospho IRE-1α, phospho PERK, Bip and XBP-1s in response to Dectin-1 stimulation.

As noted above the scope of this work was to elucidate signals that control TSLP induction secretion and not to characterise the dectin-1 induced UPR. As mentioned by reviewer 2, there are alternative activators of the ISR; we have investigated PKR as this kinase is phosphorylated following stimulation of mDC with curdlan (supplementary figure 5C). The efficacy of the PKR inhibitor was shown by its marked inhibition of PKR phosphorylation (supplementary figure 5C and manuscript lines 191-194). In support of this finding we also show no effect of the PKR inhibitor on curdlan induced TSLP expression (figure 5C-D and manuscript lines 191-194).

5. The authors observe that inhibitors of IRE-1α and PERK prevent TSLP production. Although this is an interesting finding, is this specific for TSLP? it is needed to show a cytokine not regulated by the unfolded protein response to assess specificity of the pathway.

We have shown that IL-1 secretion and mRNA expression are unaffected by PERK inhibition (figure 4a, line 208 in the manuscript, and additional supplemental figure 5F and manuscript lines 198-200). Furthermore, osteoprotegerin mRNA expression is unaffected by IRE1 inhibition, although reduced by PERK inhibition (Supplemental figure 5E and manuscript lines 198-200). Together these data show that different cytokines show different regulation by components of the UPR.

European Journal of Immunology

7. The materials and methods section is poorly written and insufficient for a manuscript in European Journal of Immunology. The information provided does not allow a clear understanding of the experimental settings. For instance, no reference is made to the pre-treatment times, particularly in experiments using blocking antibodies, inhibitors or activators. These descriptions are extremely important for appropriate interpretation of the data. In addition, no reference is made to the details (such as catalogue number) of the blocking antibody to Dectin-1.

We thank the reviewer for these useful comments and we have re-written this section as requested, adding the additional information required.

Reviewer: 3

Main points.

1 Pharmacological inhibitors studies should be complemented by siRNA approaches. Levels of cell death induced by the inhibitors should be documented.

In response to the reviewer's comments regarding using siRNA- approaches to confirm our findings, RNA interference is technically difficult in primary DC. We have extensive experience in RNA-mediated interference in macrophage/DC like cell lines (THP-1 and U937); however, these cell lines do not induce TSLP, so are not appropriate for this study. Furthermore, we now include additional data to show that the effects of the inhibitors we used are specific - see responses to reviewer 2.

2 Activation of the UPR by dectin stimulation should be documented better by determining XBP1 protein levels, impact of XBP1 targets (qPCR and protein), impact on potential RIDD targets (qPCR and protein). Indeed the level of XBP-1 mRNA splicing measured in Fig 3D are relatively low (12-18 %?) and further demonstration of XBP1 activity is required to strengthen the current observations. Next, it will be important to establish whether TSLP1 production increase results from augmenting XBP-1 expression or from an alternative implication of the RIDD activity of IRE-1. Is there an implication of the RIGI/IPS-1 pathway has suggested by Cho et al. 2013? siRNA approaches should help resolving this matter.

We have utilised a quantitative RT-PCR method for evaluating XBP1 splicing and this has been extensively validated and reflects observations at the protein level. Although the levels of XBP-1spliced (18-fold increase) are much less than those induced by chemical mediators of ER stress (100-fold increase) they are comparable to those induced by LPS (mean 10.5 fold increase, n=4 donors, data for reviewers' information). As mentioned in response to reviewer 2, despite relatively low quantities of XBP1 splicing, these quantities are biologically important. It was clearly shown by Martinon et al 2010, Nature Immunology, that activation of IRE-1 by LPS makes an important contribution to expression of the

European Journal of Immunology

cytokines, IL-6 and TNFa, even though a UPR signature equivalent to that induced by chemical inducers of ER stress was not present. The scope of this paper is to determine signals that modulate dectin1-induced TSLP from mDC and we clearly establish that dectin-1 induced IRE1 controls TSLP expression (figure 3E-F).

As stated for reviewer 2, we have modified the manuscript to indicate our emphasis on the detection of activation of proximal ER stress sensors in TSLP expression but not on the full description of the dectin-1 induced UPR.

3 As for the PERK pathway activity, the phospho-blot presented here is not of great quality. Better quality data and kinetics of eIF2a phosphorylation should be shown over several hours, matching the time of TSLP production.

We now show clearer dectin-1 induced eIF2a phosphorylation as requested (see figure 3C). Furthermore, we have performed a time course analysis of dectin-1 induced eIF2a phosphorylation: eIF2a phosphorylation was observed 1 hour post dectin-1 stimulation and was maintained at 4 hours. However, the addition of this data to the manuscript would not in our opinion be relevant to the question of how PERK activation controls TSLP induction.

4 Phosphorylation of eIF2a has been documented for monocyte-derived DCs after LPS stimulation with a different outcome that the one presented here for dectin. A comparative monitoring should be performed between dectin and TLR stimulated cells over time.

The reviewer suggests that a direct comparison of eIF2a phosphorylation by dectin-1 agonists and LPS is required, However, although interesting, such experiments would not help clarify the signals which control TSLP induction, particularly since LPS does not induce TSLP in mDC (information for reviewers, data not included in manuscript).

5 Global protein synthesis intensity should also be monitored in parallel to TSLP and other cytokines transcription as well as their translation.

This approach is useful when there is very potent ER stress and where a reduction in protein translation would indicate that the intensity of eIF2a phosphorylation is sufficient to modulate global translation rates in mDCs. Whilst potent induction of the ISR can be shown, e.g.by measuring puromycin incorporation, it is not particularly sensitive to relatively subtle activators of the ISR. Furthermore, we have evidence that GADD34 expression is dramatically induced by curdlan; the GADD34/PP1 phosphatase plays a critical role in dephosphorylation of eIF2a and enabling recovery of protein translation. This response confounds the analysis of translation rates as a useful indicator of ISR activation (information for reviewers, data not

European Journal of Immunology

shown). Most importantly, it is not clear how analysis of global translation rates would contribute to the elucidation of the mechanism of how ISR contributes to TSLP expression. Both TSLP mRNA and protein expression are significantly reduced by the inhibition of PERK (see figure 3E-F), therefore our data suggests that the ISR induces a factor required for TSLP gene expression.

6 Phospho-PERK accumulation over time should also be documented and linked to dectin stimulation and ER stress. ATF4 expression and other P-eIF2a sensitive molecules should also be monitored.

As noted previously, the aim of this paper was to address signals that control dectin-1 induced TSLP, rather than a full characterisation of the PERK controlled ISR induced through dectin-1.

7 No evaluation on IL-1 expression and its processing is provided in the current version of the manuscript. It will be important to establish the impact of Dectin 1 and UPR pathways on inflammasome activity and IL-1 processing.

To clarify, we have established that PERK inhibition has no effect on IL-1 secretion and therefore no role in inflammasome activation (figure 4A). Furthermore, PERK inhibition has no effect on IL-1β mRNA expression (see supplementary figure 5F), therefore we suggest there is no role of the ISR activation in IL-1 expression. We do suggest however that another proximal sensor of the ER stress response, IRE-1, plays a role in IL-1 expression, since IRE1 inhibition reduces IL-1 secretion.

8 An attempt should be made to have the demonstration that UPR is induced significantly upon Candida infection or dectin stimulation in DCs or macrophages in vivo, to give physiological relevance to the findings.

Clearly evidence of UPR induction by Candida infection in vivo would be of great interest, but we suggest that this work is beyond the scope of this manuscript.

<u>Second Editorial Decision – 21 May 2015</u>

Dear Mr. Elder,

Thank you for submitting your revised manuscript ID eji.201545537.R1 entitled "TSLP production by dendritic cells is modulated by IL-1β and components of the endoplasmic reticulum stress response" to the European Journal of Immunology. Your manuscript has been re-reviewed and the comments of the referees are included at the bottom of this letter.

Please note that two of the reviewers still thought that your manuscript would greatly benefit from the addition of siRNA experiments. We have noticed that in your response letter you already explained that siRNA experiments are difficult in primary DCs and we agree on that point. But since both of the reviewers and also our Executive Commitee agree that those experiments would be helpful, we wanted to know whether difficult means "impossible" or just difficult. Therefore, if you see any chance to add siRNA exeriments we would really encourage you to do so.

The journal does not encourage multiple rounds of revision and you should fully address the concerns of the referee in this final round of revision. Should you disagree with any of the referees' concerns, you should address this in your point-by-point response and provide solid scientific reasons for why you will not make the requested changes.

You should also pay close attention to the editorial comments included below. **In particular, please edit your figure legends to follow Journal standards as outlined in the editorial comments. Failure to do this will result in delays in the re-review process.**

Please note that submitting a revision of your manuscript does not guarantee eventual acceptance, and that your revision will be re-reviewed by the referees before a decision is rendered.

If the revision of the paper is expected to take more than three months, please inform the editorial office. Revisions taking longer than six months may be assessed by new referee(s) to ensure the relevance and timeliness of the data.

Once again, thank you for submitting your manuscript to European Journal of Immunology and we look forward to receiving your revision.

Yours sincerely,
Katharina Schmidt

On behalf of Prof. Caetano Reis e Sousa

Dr. Katharina Schmidt
Editorial Office
European Journal of Immunology
e-mail: ejied@wiley.com
www.eji-journal.eu

# Peer review correspondence

European Journal of Immunology

********************

Reviewer: 1

Comments to the Author
The authors addressed some of the comments of the reviewers, the manuscript is improved. A major criticism that remains is that the study is based mostly on chemical inhibitors, and confirmations with siRNA or other means is not provided. The authors addressed this by referring that these inhibitors are comonly used in the literature and that gene expression manipulation is difficult to set up in their system.

Reviewer: 2

Comments to the Author
This reviewer is satisfied with the authors's response and the added experiments. The manuscript has improved in quality and clarity and is now suitable for publication in the European Journal of Immunology.

Reviewer: 3

Comments to the Author
I still believe that an attempt to perform siRNA in MoDCs should be provided to demonstrate the implication of XBP1, PERK and Casp1 in this process. The mechanisms linking the UPR to TSLP production is still really unclear and should be better defined.

<u>Second revision – authors' response – 2 October 2015</u>

We would like to thank the reviewers and executive committee for their additional comments on our manuscript entitled 'TSLP production by dendritic cells is modulated by IL-1β and the endoplasmic reticulum stress response'.

We have modified the figure legends to comply with EJI standards as requested.

We do acknowledge that siRNA knockdown of the ER stress sentinels IRE-1 and PERK would further strengthen our evidence that ER stress signals contribute to the expression of TSLP.

We have subsequently invested considerable time in utilising a siRNA approach to knockdown these molecules in human dendritic cells. We developed an approach that utilised a double delivery of siRNA

European Journal of Immunology

that did result in the knockdown of PERK gene expression but this was not sufficient to significantly impact on the activation of downstream effectors in the integrated stress response. We have been in consultation with David Ron's laboratory (CIMR Cambridge) which specialises in ER stress and they have advised us that the long half-life of the PERK protein does indeed preclude siRNA approaches to ablate PERK activity. A similar result and difficulty was found using IRE-1 siRNA as we were not able to significantly impact on the activation of downstream effectors despite IRE-1 knockdown. Although we do have shRNA that effectively target IRE-1, this reagent can only be used in cell lines, and none of these lines secrete TSLP in response to dectin 1 agonists. We do however have an IRE-1 fl/fl x LysM cre mouse which results in the loss of IRE-1 in the macrophage lineage (BMBMs) but we found that the expression of TSLP in BMDMs following dectin 1 agonist stimulation was not detectable in wildtype mice.

We think that despite the lack of siRNA approaches in this manuscript we provide strong evidence that ER stress signals are required for TSLP expression.
1. The use of ER stress inducing chemicals that induce ER stress via independent pathways, strongly enhance TSLP expression in dendritic cells stimulated with dectin 1 agonists.
2. Dectin 1 agonists alone induce the unfolded protein response (UPR) as demonstrated by XBP-1 splicing and eIF2α phosphorylation.
3. The use of well validated chemical inhibitors of IRE-1 (and PERK reduce the expression of TSLP induced by dectin 1 agonists.
Furthermore, given that alternative eIF2α kinases could be inducing the integrated stress response (ISR), we pinpoint ER stress and PERK as the inducer of the ISR, this is clearly shown by the loss of eIF2α phosphorylation in the presence of the PERK inhibitor.

Additional responses to reviewers.
Reviewer 3 states that a casp1 knockdown approach should be performed in human DCs to provide evidence that caspase1 is required for IL-1β production in response to dectin 1 agonists.
We feel that we have provided sufficiently strong evidence for this conclusion. In addition to the use of caspase specific inhibitors we provided additional data using casp 1 KO mice. We were able to show that Casp1 was required for dectin 1 agonist induction of IL-1β in murine BMDCs. Most importantly, this finding is not without precedent, several high impact publications have shown an essential role for caspase-1 in dectin-1 induced IL-1β (Gross et al Nature 2009. 459: 433-436.) and more recently by Ganesan et al (J Immunol 2014. 193: 2519-2530).

Given that we show the novel data that TSLP is highly dependent on IL-1β signalling, we conclude that TSLP secretion is indirectly dependent on casp1.

# Peer review correspondence

European Journal of Immunology

Third Editorial Decision – 22 October 2015

Dear Prof. Gaston, Mr. Elder,

It is a pleasure to provisionally accept your manuscript entitled "TSLP production by dendritic cells is modulated by IL-1β and components of the endoplasmic reticulum stress response" for publication in the European Journal of Immunology. For final acceptance, please follow the instructions below and return the requested items as soon as possible as we cannot process your manuscript further until all items listed below are dealt with.

Please note that EJI articles are now published online a few days after final acceptance (see Accepted Articles: http://onlinelibrary.wiley.com/journal/10.1002/(ISSN)1521-4141/accepted). The files used for the Accepted Articles are the final files and information supplied by you in Manuscript Central. You should therefore check that all the information (including author names) is correct as changes will NOT be permitted until the proofs stage.

We look forward to hearing from you and thank you for submitting your manuscript to the European Journal of Immunology.

Yours sincerely,
Karen Chu

on behalf of Prof. Caetano Reis e Sousa

Dr. Karen Chu
Editorial Office
European Journal of Immunology
e-mail: ejied@wiley.com
www.eji-journal.eu
